# Beyond recidivism: changes in health and social service involvement following exposure to drug treatment court

**DOI:** 10.1186/s13011-015-0038-x

**Published:** 2015-10-31

**Authors:** Stefanie N. Rezansoff, Akm Moniruzzaman, Elenore Clark, Julian M. Somers

**Affiliations:** Faculty of Health Sciences, Simon Fraser University, 8888 University Drive, Burnaby, British Columbia V5A 1S6 Canada; British Columbia Corrections, PO Box 9242, STN PROV GOV, Victoria, British Columbia V8W 9 J2 Canada

**Keywords:** Drug treatment court, Substance use treatment, Drug-involved offenders, Concurrent disorders, Social determinants of health

## Abstract

**Background:**

The majority of Drug Treatment Court (DTC) research has examined the impact of DTCs on criminal recidivism. Comparatively little research has addressed the association between DTC participation and engagement with community-based health and social services. The present study investigated changes in participant involvement with outpatient healthcare and income assistance within a DTC cohort. We hypothesized that involvement with community-based (outpatient) health and social services would increase post-DTC participation, and that service levels would be higher among program graduates and offenders with histories of co-occurring mental and substance use disorders.

**Methods:**

Participants were 631 offenders at the DTC in Vancouver, Canada (DTCV). Administrative data representing hospital, outpatient medical care, and income assistance were examined one-year pre/post program to assess differences over time. Generalized estimating equations were used to investigate the association between changes in service use and program involvement. We also examined the relationship between level of service use and offender characteristics.

**Results:**

Members of the cohort were disproportionately Aboriginal (33 %), had been sentenced 2.7 times in the 2 years preceding their index offence, and 50 % had been diagnosed with a non substance-related mental disorder in the five years preceding the index offence. The mean number of outpatient services post DTCV was 51, and the mean amount of social assistance paid was $5,897. Outpatient service use increased following exposure to DTCV (Adjusted Rate Ratio (ARR) = 1.45) and was significantly higher among women (ARR = 1.47), program graduation (ARR = 1.23), and those previously diagnosed with concurrent substance use and mental disorders (ARR = 4.92). Overall, hospital admissions did not increase post-program, although rates were significantly higher among women (ARR = 1.76) and those with concurrent disorders (ARR = 2.71). Income assistance increased significantly post program (ARR = 1.16), and was significantly higher among women (ARR = 1.03), and those diagnosed with substance use disorders (ARR = 1.42) and concurrent disorders (ARR = 1.72).

**Conclusions:**

These findings suggest that the DTCV was a catalyst for increased participant engagement with community health and social supports, and that rates of service use were consistently higher among women and individuals with concurrent disorders. Research is needed to investigate the potential link between health and social support and reductions in recidivism associated with DTCs.

## Background

An overwhelming majority of peer-reviewed literature finds that drug treatment courts (DTCs) achieve their mandate of reducing the likelihood of substance-related criminal recidivism. These specialized courts hinge on the coordinated response of a dedicated team of judges, prosecutors and defence counsel, probation authorities and other key players. DTCs adjudicate dockets of selected, non-violent substance-dependent offenders who have agreed to court-monitored substance use treatment in lieu of traditional administration of justice.

The most recent meta-analysis assessing the effectiveness of DTCs on recidivism (published in 2012), found “the average effect of participation … analogous to a drop in recidivism from 50 to 38 %” ([1]; p.60) and that “… these effects appear to last at least three years post drug court entry” ([1]; p.68)). Subsequent individual studies affirm these findings [2, 3], and also suggest that DTCs are more effective than probation at preventing recidivism among drug-involved individuals [4].

Since their inception in 1989, DTCs have proliferated and transformed the way criminal justice systems deal with drug-involved offenders throughout the Western hemisphere [5], (including the Caribbean), as well as in Europe and Australasia [6]. According to the National Institute of Justice, there are more than 3,400 drug courts throughout the US [7], while the Canadian Association of Drug Treatment Court Professionals reports 10 DTCs in Canada [8]. Prior to the completion of the first Canadian outcome studies on DTCs, some scholars had raised concerns related to their ethics and effectiveness [9, 10]. To promote consistency, national and international organizations have provided guidelines for the operation of DTCs, with a number of key components in common, *extending beyond the integration of substance use treatment with justice system case processing*. These include timely intervention for eligible clients, a non-adversarial approach to achieving abstinence, ongoing judicial monitoring, evaluation and intervention, and continuous, coordinated support from court, public sector and community-based personnel [11].

The current paradigm shift to evidence-based justice policy has been associated with new developments in our understanding of the causes of criminal behaviour and recidivism [12]. Income inequality [13], social exclusion [14] and poor health [15] are inextricably linked with crime and criminal behaviour. Social determinants, including unstable or precarious housing, lack of education and unemployment, also contribute to illicit drug use [16], and to the aetiology of mental and substance use disorders [17]. Substance use disorders, particularly when they co-occur with mental disorders, are significantly associated with criminal behaviour [18, 19]. And while some evidence indicates that preventing recidivism can involve addressing the symptoms of these disorders [20], in the majority of cases, effective prevention efforts *also* require individually targeted efforts addressing broader social determinants of health [21, 22].

Somewhat ironically - in the midst of the current paradigm shift to evidence-based justice policy – the drug court movement occurred *prior* to the development of a strong evidence base in its favour [1]; perhaps as a pragmatic response to unmanageably high rates of recidivism among burgeoning numbers of drug-related offenders. Nonetheless, the success of DTCs strengthens the notion that social determinants of health should be routinely addressed *alongside symptoms*, among offenders with mental and/or substance use disorders [23].

DTCs now encompass a wide variety of dockets targeted at specific drug-involved populations. These include veterans [24], families and juveniles [25], offenders with co-morbid mental and substance use disorders [26], individuals arrested for driving ‘under the influence’ or repeated ‘driving while intoxicated’ [27] and Aboriginal tribal drug courts [28] (also known as Healing to Wellness Courts). These variations aim to address the heterogeneous needs and social contexts of drug-involved offenders, and are predominantly found in the US. In both the US and Canada, however, it has become clear that understanding the overall impact of DTCs on outcomes *other than drug use and criminal behaviour* is an important component to reducing recidivism across a wide variety of DTC subpopulations [29].

A body of research attests to the importance [30] and effectiveness [31–33] of community-based mental health treatment for offenders. New research suggests that while symptoms play a limited role in criminal risk assessment, their interaction with factors such as social determinants of health “…potentiate general risk factors … [and] become part of the criminogenic story” [23; p. 920]. With DTCs effectively rooted in this philosophy, literature demonstrates that they effectively reduce recidivism [1] in a dose–response manner, [2, 34], and particularly among those who graduate from the program [35]. Hence the current focus of research on program retention and completion [see 4].

Notwithstanding a limited literature demonstrating positive psychosocial outcomes among drug court participants [36], most existing DTC research is targeted to understanding the effects of DTC engagement on recidivism (as the outcome variable). Additional research is needed in order to investigate whether DTCs stimulate engagement with health and social services, and thereby may help address offender needs apart from substance use, such as physical and mental illness and precarious housing.

### The Drug Treatment Court of Vancouver (DTCV)

The DTCV began operations on December 4th, 2001, as the second drug treatment court to open in Canada. It is located in the Provincial Court facility of Vancouver’s Downtown Eastside, infamously known as one of Canada’s poorest neighbourhoods. The Downtown Eastside consists of a 10 square block area with a chaotic street scene, well-documented drug and mental health related problems, high crime rates and prevalent homelessness [37]. Although the court began as a pilot project to serve the needs of offenders residing in this neighbourhood, its catchment area has expanded substantially to include referrals from court jurisdictions in several cities surrounding Vancouver.

Now one of the largest DTCs in Canada, it acts as both provider and broker of professional supports and substance use treatment, and coordinates diverse resources to address interdependent medical, social and legal issues faced by individuals who commit minor offences as a result of their substance use. Offenders are also referred to the DTCV by provincial courts, defence counsel and Crown counsel, and commonly present complex health, housing and vocational challenges alongside their involvement with substance use and crime.

During the first few years following inception, the DTCV’s in-house medical resources were limited to a single addiction treatment provider. In 2006 the program was substantially revised and expanded to provide a broader range of treatment targets, including mental health, general health and health promotion, (in addition to the main focus on addiction treatment). These services are now delivered by a dedicated DTCV team, with representatives from the major government agencies responsible for health and social services.

Under the revised model, nurses, a psychologist, addictions counselors, housing and social assistance support workers, and an Aboriginal liaison deliver treatment, while participants’ progress through the program is closely monitored by court staff, probation officers, defence and Crown counsel and DTCV dedicated judges. Members of the DTCV team function in a non-adversarial manner to carry out case planning, monitoring, and transitioning to community-based care. Judges join a collegial network of their peers and participate in national and international conferences as well as informal education opportunities. Team members collaborate to address the complex concomitant needs of drug-involved offenders in Vancouver’s downtown, including addictions, health and psychiatric care, as well as life skills training, education, housing, financial assistance and leisure activities.

Completion of the program, or ‘graduation’ requires a minimum of 14 months of drug treatment supervised by a DTCV judge who reviews each client’s progress through consecutive phases of the program. Additional details of the court’s activities are included in previous publications [38, 39].

Previous research on the DTCV has demonstrated that compared to a matched group of offenders, those assigned to the DTCV had significantly lower rates of re-offending [38]. Further research demonstrated that the impact of the DTCV on recidivism was significantly higher among women and Aboriginal offenders, and that there were no significant differences in outcome between those diagnosed with co-occurring mental disorders and those without [39]. The purpose of the present analysis was to examine whether participants of the DTCV exhibited changes in health and social service use following program involvement, and whether levels of service use were also associated with length of exposure to the program as well as to offender characteristics.

Our primary hypothesis was that involvement with community (outpatient) health and social services would increase post-DTCV participation. We also hypothesized that participants diagnosed with mental disorders or concurrent disorders would exhibit a relatively high level of service use.

## Methods

### Ethics statement

This study involved the analysis of de-identified records and was approved by the Research Ethics Board of Simon Fraser University and the applicable research committees, privacy committees, and offices of the Assistant Deputy Ministers within the Government of British Columbia, Canada. Due to the anonymous nature of the data neither written nor verbal consent was possible.

### Data sources

We examined de-identified linked administrative data spanning three provincial government ministries: Justice; Health Services; and Social Development & Social Innovation. Data from the contributing ministries comprise a relatively complete inventory of the health, justice, and income assistance services used by members of the British Columbia (BC) population. The completeness of these data reflects the central organizational and funding role provided by the provincial government in the administration of these various program areas. Record linkage followed a multi-stage probabilistic algorithm and resulted in an 89 % match rate [18].

### Participants

Participants were included if they were enrolled in the DTCV between its inception on December 4th, 2001 and March 31st, 2011, and regardless of their status at the time of program completion, (i.e., using an intent to treat design). We restricted inclusion to individuals who exited the DTCV by March 31st, 2011, ensuring at least 12 months of follow-up data for each participant. Individuals who died during the follow-up period were excluded. Individuals with less than one year follow-up were also excluded. Follow up started on exit from the DTCV. Graduation, withdrawal and termination from the DTCV were each considered ‘exit’ from the program.

Using an intent-to-treat approach, post-treatment program effects included all individuals regardless of status at the time of program completion (i.e., graduated, withdrew or discharged). For the purposes of pre/post analysis, data were obtained for one year for both the pre and post periods of the DTCV intervention.

The age of participants was calculated at the time of enrolment in DTCV, and was treated as a continuous variable in the analysis. Self-reported gender was obtained from Ministry of Justice data at the date of first sentence. Ethnicity was limited to three groups: White, Aboriginal (Aboriginal, Métis, First Nations, Native or Inuit) and Other (Asian, Black, East Indian, Hispanic or other category). In order to assess the impact of changes in the DTCVs resources and design over time (additional details below), periods of enrolment were defined as: prior to 2005; between 2005 and 2007, and after 2007.

### Variables

Our models included independent variables related to offender risk and need: age at enrolment; gender^a^; ethnicity; formal education level; and offence history. Our dependent variables were selected based on their relevance to the prevention of acute illness, improved access to community health services, and increased access to financial supports to which individuals are entitled. We examined overall amounts of financial assistance received as well as the portion of support provided to secure shelter and thereby reduce homelessness or precarious housing. Health-related variables included provincial Medical Services Plan (MSP) outpatient services and acute hospitalizations^b^.

MSP billing data represent all medically required physician and related laboratory services (e.g., monitoring of reactions to and titration of medications) provided to citizens in BC. MSP data were included due to their sensitivity as a measure of physician engagement and continuity of community care; recognizing that offenders with high needs frequently experience barriers to community-based care for general health concerns, mental health treatment, and substance use treatment. Medically necessary hospital services are also universally provided to all BC residents. Acute hospitalizations were included to examine whether the DTCV promoted an overall change in the rate of acute health concerns requiring admission. Social assistance payments (CAD$) were selected as indicators of the amount of support received by participants, (including funds for shelter).

### Diagnostic measures

Mental disorder diagnoses were based on the ninth edition of the International Classification of Diseases (ICD). All diagnoses were administered by physicians (family physicians and psychiatrists) practicing under the Medical Services Plan (MSP). All disorders were included within the ICD range of 290–319 (Mental Disorders). Substance use disorders (SUDs) were identified using the 3 digit codes of 291, 292, 303, 304, and 305. Non-substance-related mental disorders (NSMDs) consisted of all other codes within the range identified. Individuals diagnosed with at least one NSMD and at least one SUD were identified as having dual diagnosis status.

Following the procedures outlined above, (and previous analyses involving the BC offender population [18]), four non-overlapping groups were established based on the diagnosed prevalence of mental disorders in the 5-year period prior to entering the DTCV, and using the ICD codes described above. The resulting groups were labeled as follows: 1) No diagnosis; 2) NSMD; 3) SUD, and 4) Both NSMD and SUD.

### Analyses

Descriptive statistics were used to characterize the sample (means and standard deviations for continuous variables and proportions for categorical variables). We compared variables between groups using parametric tests (student t-test for continuous variables) and non-parametric (Pearson’s chi-square test for categorical variables), as appropriate.

Generalized Estimating Equations (GEE) were used to estimate changes over time (post-treatment vs. pre-enrolment period) for the outcome variables (MSP services, acute hospital admissions and social assistance payments) among DTCV participants. Negative binomial models (NBR; negative binomial distribution with log link) were selected due to the over-dispersion and count nature of outcome data, and for better goodness of fit statistics relative to Poisson regression.

Exchangeable correlation structure and a robust method were chosen to control for dependency over time between pre and post enrolment periods, and to estimate standard errors for the parameters, respectively. In addition, socio-demographic variables (e.g., age at enrolment, gender, ethnicity, education level, offence history, etc.) were included in the multivariable regression analysis to control for the effects of potential confounders. All costs were adjusted for inflation and converted to 2012 Canadian dollars based on rates obtained from the Bank of Canada [40]. Payments were rounded to the nearest integer.

An alpha level 0.05 was used to assess the significance of variables of interest. All reported *p*-values were two-sided. Rate Ratios (RR) obtained from the NBR models were reported as effect sizes. Missing values for covariates ranging from zero to 4 % (i.e., age & gender: 0 %, ethnicity: 1 % and education: 4 % were excluded from analyses.

IBM SPSS Statistics 22 (IBM Corp. Released 2013. IBM SPSS Statistics for Macintosh, Version 22.0. Armonk, NY: IBM Corp.) and STATA 13 (StataCorp. 2013. *Stata Statistical Software: Release 13*. College Station, TX: StataCorp LP) were used to conduct these analyses.

## Results

Since its inception in December 2001, a total of 836 participants were enrolled into the DTCV until March 31st, 2011. Among them, 205 individuals were excluded for any of the following reasons: missing administrative data (*n* = 43); active program status (*n* = 77); less than 1-year follow up period (*n* = 70); and deceased during the 1-year follow up period (*n* = 15). A total of 631 individuals were available for inclusion in the analysis, and were between 18 and 67 years of age. Table 1 presents the socio-demographic characteristics and details of any previous history with the DTCV for the cohort.

**Table 1:** Socio-demographic, diagnostic and sentence-related characteristics of DTCV Participants (*n* = 631)

Variables	DTCV Participants (*n* = 631)
	n (%)
Age at enrolment in years	
Mean (SD)	34.9 (9.0)
Gender	
Men	402 (64)
Women	229 (36)
Ethnicity	
White	338 (54)
Aboriginal	204 (33)
Other	80 (13)
Education level	
Grade 9 or less	94 (15)
Grade 10/11	261 (43)
Grade 12	192 (32)
Vocational /University	60 (10)
Program status (last episode)	
Graduated	152 (24)
Didn’t graduate	479 (76)
Year of enrolment (first episode)	
Before 2005	245 (39)
Between 2005 and 2007	230 (36)
After 2007	156 (25)
Number of admissions	
Single	513 (81)
Multiple	118 (19)
History of mental disorder (last 5 years)	
No mental disorder	180 (29)
NSMD only	57 (9)
SUD only	135 (21)
Both NSMD & SUD	259 (41)
Number of sentences (last 2 years)	
Mean (SD)	2.7 (4.3)
Number of drug related offences (last 2 years)	
Mean (SD)	1.1 (1.5)
Number of breach offences (last 2 years)	
Mean (SD)	0.7 (1.5)
Number of property offences (last 2 years)	
Mean (SD)	1.1 (2.4)

*DTCV* Drug Treatment Court of Vancouver, *NSMD* non-substance related mental disorders, *SD* standard deviation, *SUD* substance use disorders

Mean age at enrolment was 35 years. The majority of participants were White (54 %) and men (64 %). Fully one third of participants were of self-reported Aboriginal ethnicity. Overall, participants attained a relatively low level of education, with 32 % having completed high school and 10 % with more advanced education. About one-quarter (24 %) of the sample fulfilled all necessary requirements for graduation and 20 % were admitted to the court more than once during the period of analysis.

Medical histories indicated that nearly two thirds (62 %) of the sample had been diagnosed with and received treatment for a substance use disorder within the five years prior to entering DTCV. The majority of these individuals also had co-occurring mental disorders. The average number of sentences in the two years prior to enrolment (2.7) was also high. Roughly half of these sentences were related to property offences, while half were attributed to drug-related or breach offences. Comparison between the one-year pre versus one year post treatment (see Table 2) indicates a significant (*p* < 0.001) increase in the number of Medical Services Plan (MSP) visits, from a mean of 39.9 to 51.1 per person. The amount of social assistance received also increased significantly from $5,130 to $5,897 (Canadian dollars) over the same period (*p* < 0.001). There was no significant change in the number of acute hospital admissions.

**Table 2:** Service use comparisons (1 year) pre and (1 year) post-treatment (*n* = 631)

	Pre-program period Mean (SD)	Post-program period Mean (SD)	Pre-post Mean (SD)	*P* value^a^
# of MSP services	39.9 (59.3)	51.1 (65.1)	−11.2 (60.6)	<0.001
# of acute hospitalizations	0.3 (0.9)	0.3 (0.8)	0.0 (0.9)	0.931
Social assistance payments ($CAD)	5130 (4367)	5897 (4767)	−767 (4271)	<0.001

*MSP* medical service plan, *SD* standard deviation

^a^ -p values were obtained from paired t test (df = 630, two tailed)

GEE analysis was conducted to examine the independent associations between the program effect, and health and social service use in the post period. Table 3 presents Unadjusted and Adjusted Rate Ratios (URR and ARR) and significance values related to Medical Services Plan (MSP) use. Adjusted models indicate that time (one year pre versus one year post DTCV), younger age, program graduation and gender (women) were associated with significantly higher rates of MSP service use. Compared to the reference group, MSP services were significantly greater among participants with a diagnosed mental or substance use disorder. Use of medical services was particularly marked among those diagnosed with concurrent disorders.

**Table 3:** GEE analysis estimating post-treatment effect on MSP use (*n* = 631)

Variables	Unadjusted rate ratio (95 % CI)	*P* value	Adjusted rate ratio (95 % CI)	*P* value
Post-treatment period (vs. pre-treatment)	1.28 (1.15, 1.42)	<0.001	1.45 (1.28, 1.64)	<0.001
Age at enrolment (per year)	1.03 (1.02, 1.04)	<0.001	1.03 (1.02, 1.04)	<0.001
Women	1.22 (1.01, 1.47)	0.044	1.47 (1.20, 1.81)	<0.001
Ethnicity				
White	1.57 (1.19, 2.07)	0.001	1.06 (0.80, 1.41)	0.679
Aboriginal	1.28 (0.95, 1.74)	0.108	0.98 (0.72, 1.34)	0.915
Other	Reference		Reference	
Education level				
Grade 9 or less	0.95 (0.67, 1.37)	0.797	0.86 (0.60, 1.23)	0.405
Grade 10/11	0.94 (0.69, 1.29)	0.704	0.87 (0.64, 1.18)	0.372
Grade 12	0.82 (0.60, 1.14)	0.243	0.70 (0.51, 0.95)	0.024
Vocational /University	Reference		Reference	
Graduated (vs. didn’t graduate)	1.26 (1.03, 1.54)	0.028	1.23 (1.01, 1.50)	0.045
Year of enrolment				
Before 2005	Reference		Reference	
Between 2005 and 2007	1.09 (0.87, 1.37)	0.452	1.06 (0.87, 1.30)	0.544
After 2007	1.42 (1.12, 1.80)	0.003	1.22 (0.95, 1.58)	0.114
Multiple admissions (vs. single)	0.83 (0.66, 1.04)	0.107	1.04 (0.83, 1.30)	0.713
Mental disorder in last 5 years				
No mental disorder	Reference		Reference	
NSMD only	2.46 (1.74, 3.48)	<0.001	2.10 (1.51, 2.91)	<0.001
SUD only	3.48 (2.60, 4.66)	<0.001	3.42 (2.57, 4.56)	<0.001
Both NSMD & SUD	4.98 (3.88, 6.39)	<0.001	4.92 (3.83, 6.32)	<0.001
# of sentences in last 2 years (per sentence)	0.96 (0.94, 0.99)	0.001	0.98 (0.95, 1.00)	0.023

Table 4 presents results associated with DTCV rates of acute hospital admissions. Program graduates had a significantly lower risk of hospitalization, while gender (women) was associated with a significantly greater risk. Participants diagnosed with concurrent substance use and mental disorders exhibited a significantly higher rate of acute hospital admissions.

**Table 4:** GEE analysis estimating post-treatment effect on acute hospital admissions (*n* = 631)

Variables	Unadjusted rate ratio (95 % CI)	*P* value	Adjusted rate ratio (95 % CI)	*P* value
Post-treatment period (vs. pre-treatment)	1.01 (0.79, 1.29)	0.931	1.05 (0.82, 1.35)	0.712
Age at enrolment (per year)	1.00 (0.98, 1.01)	0.704	1.01 (0.99, 1.03)	0.261
Women	2.04 (1.43, 2.90)	<0.001	1.76 (1.23, 2.51)	0.002
Ethnicity				
White	0.99 (0.59, 1.65)	0.978	0.74 (0.44, 1.24)	0.251
Aboriginal	1.55 (0.91, 2.64)	0.106	1.10 (0.64, 1.89)	0.725
Other	Reference		Reference	
Education level				
Grade 9 or less	1.99 (1.06, 3.73)	0.033	1.39 (0.72, 2.68)	0.324
Grade 10/11	1.28 (0.71, 2.29)	0.411	0.97 (0.53, 1.77)	0.929
Grade 12	1.27 (0.66, 2.44)	0.468	1.05 (0.55, 2.00)	0.893
Vocational /University	Reference		Reference	
Graduated (vs. didn’t graduate)	0.52 (0.32, 0.87)	0.012	0.55 (0.32, 0.93)	0.025
Year of enrolment				
Before 2005	Reference		Reference	
Between 2005 and 2007	1.19 (0.76, 1.86)	0.452	1.40 (0.93, 2.10)	0.107
After 2007	1.21 (0.76, 1.94)	0.424	1.41 (0.88, 2.27)	0.152
Multiple admissions (vs. single)	1.15 (0.78, 1.69)	0.484	1.35 (0.89, 2.03)	0.155
Mental disorder in last 5 years				
No mental disorder	Reference		Reference	
NSMD only	1.21 (0.64, 2.27)	0.564	1.02 (0.55, 1.88)	0.962
SUD only	1.79 (1.08, 2.97)	0.023	1.39 (0.84, 2.29)	0.201
Both NSMD & SUD	2.88 (1.82, 4.56)	<0.001	2.71 (1.70, 4.34)	<0.001
# of sentences in last 2 years (per sentence)	0.98 (0.92, 1.04)	0.442	0.98 (0.92, 1.05)	0.561

*CI*, confidence interval, *GEE*, generalized estimating equation, *MSP* medical service plan, *NSMD* non-substance related mental disorders, *SUD* substance use disorder

Time, age, graduation and gender (women) were each associated with significantly higher rates of social assistance payments, as was enrolment after 2007 (see Table 5). Participants with concurrent disorders were associated with significantly higher rates of payments, while the number of prior sentences in the preceding 2 years was associated with a lower level of social assistance.

**Table 5:** GEE analysis estimating post-treatment effect on total social assistance payments (*n* = 631)

Variables	Unadjusted rate ratio (95 % CI)	*P* value	Adjusted rate ratio (95 % CI)	*P* value
Post-treatment period (vs. pre-treatment)	1.15 (1.08, 1.22)	<0.001	1.16 (1.08, 1.25)	<0.001
Age at enrolment (per year)	1.03 (1.02, 1.04)	<0.001	1.03 (1.02, 1.04)	<0.001
Women	1.06 (0.94, 1.20)	0.341	1.17 (1.02, 1.35)	0.022
Ethnicity				
White	1.21 (1.00, 1.46)	0.050	1.00 (0.81, 1.23)	0.995
Aboriginal	1.17 (0.96, 1.44)	0.125	1.14 (0.91, 1.42)	0.264
Other	Reference		Reference	
Education level				
Grade 9 or less	0.90 (0.73, 1.12)	0.342	0.89 (0.70, 1.13)	0.353
Grade 10/11	0.88 (0.74, 1.05)	0.154	0.86 (0.70, 1.06)	0.153
Grade 12	0.86 (0.72, 1.03)	0.093	0.80 (0.65, 0.99)	0.035
Vocational /University	Reference		Reference	
Graduated (vs. didn’t graduate)	1.20 (1.06, 1.35)	0.003	1.14 (1.00, 1.30)	0.055
Year of enrolment				
Before 2005	Reference		Reference	
Between 2005 and 2007	1.17 (1.01, 1.35)	0.038	1.08 (0.94, 1.24)	0.301
After 2007	1.48 (1.30, 1.68)	<0.001	1.37 (1.19, 1.57)	<0.001
Multiple admissions (vs. single)	0.91 (0.79, 1.04)	0.163	1.14 (0.97, 1.35)	0.105
Mental disorder in last 5 years				
No mental disorder	Reference		Reference	
NSMD only	1.41 (1.13, 1.75)	0.002	1.24 (0.98, 1.57)	0.075
SUD only	1.51 (1.26, 1.80)	<0.001	1.42 (1.20, 1.69)	<0.001
Both NSMD & SUD	1.80 (1.54, 2.12)	<0.001	1.72 (1.47, 2.01)	<0.001
# of sentences in last 2 years (per sentence)	0.97 (0.96, 0.98)	<0.001	0.98 (0.96, 0.99)	0.005

*CI* confidence interval, *GEE* generalized estimating equation, *NSMD* non-substance related mental disorders, *SUD* substance use disorders

## Discussion

DTCV participants demonstrated increased engagement with community-based health and social services following their involvement with the program. Higher levels of overall service use were consistently associated with offenders who were diagnosed with concurrent substance use and mental disorders prior to the index offence. Our results confirm that the DTCV targeted offenders with interdependent and often mutually exacerbating medical, psychiatric, social and legal issues. Although DTCV graduates were older, more likely to be men and to have achieved a higher level of education, it is important to note that our hypotheses did not include examination of the interaction between graduation status and DTCV involvement.

The DTCV team initiates the receipt and coordination of resources intended to meet the needs of offenders, including medical care, mental health services, housing support, and income assistance alongside substance use treatment. Our results build on previous findings indicating that the DTCV reduces the risk of recidivism [38], and suggest that changes in health and social domains coincide with the impact on offending. Further research is required to examine the relationships between health, social and substance use interventions with criminal recidivism. Participants in the DTCV cohort were selected because they faced conspicuous problems involving drug use and crime. However, only 62 % of participants had been previously diagnosed with substance dependence in the public healthcare system. This suggests that the court may have served to initiate treatment for a subset of individuals.

Notably, one third of DTCV participants self-identified as Aboriginal, compared to approximately 5 % of the British Columbia provincial population who self-identified as Aboriginal during the study period^c^. This also represents a considerably higher proportion of Aboriginal persons than previously reported among offenders sentenced though the BC Provincial court facilities where the DTCV is located in the Downtown Eastside of Vancouver (17 %) [38], as well as within the general population of offenders in British Columbia (19 %) [18]. Notwithstanding previous research findings that the DTCV is comparatively more effective at reducing recidivism among Aboriginal participants [39], the over-representation of Aboriginal people in the current study is in keeping with their disproportionate and widely recognized involvement with the criminal justice system [41–43].

The complexity of need among members of the cohort was illustrated by the finding that 50 % of the sample had been diagnosed with at least one non substance-related mental disorder, in addition to their problems concerning substance use. This finding raises concern, given that co-occurring substance use and mental disorders have been shown to be highly predictive of criminal behaviour and recidivism [18, 44], and represent criminogenic needs that are amenable to change through appropriate intervention [45–47].

Criminal history included nearly 3 sentences *per participant* in the two years prior to enrolment in the DTCV. This relatively high rate of prior convictions may be related to the effectiveness of the DTCV, as evidence indicates that previous convictions are predictive of treatment effects. In their 2005 meta-analysis of DTC effectiveness, Lowenkamp, Holsinger & Latessa concluded: “ … the effectiveness of drug courts *doubled* [emphasis added] when 50 % or more of the drug court participants had a prior record” ([48]; p.28). Further research is needed to investigate this interaction and improve DTC outcomes regardless of offenders’ prior criminal history.

Notably, medical service use increased significantly in the year following involvement with the DTCV (to 51 encounters per person) compared to the year preceding enrolment (40 encounters per person), although there were no significant changes in the overall number of acute hospital admissions between the same two periods. While this suggests that participant health-related needs and conditions were being more actively managed by non-urgent community-based (outpatient) care, the increase in community-based health care use was not evenly distributed throughout the cohort. Women and participants with co-occurring disorders exhibited a significantly *higher* rate of acute hospital admissions in the post period, indicating that access to community-based (outpatient) care may still require bolstering for these two sub-groups.

The amount of social assistance and shelter payments received also increased significantly in the post-treatment period. This may be due, in part, to substantial staff and programing expansions undertaken in 2006. Following these changes, it is possible that team members were more likely to facilitate engagement with income assistance – either because of better staffing and/or because they saw a direct link between income and the ongoing need for health services. The provision of financial support is essential for shelter, clothing, and other basic needs. However, it may also be important to ensure that funds are used appropriately, as some research suggests that income assistance received by residents of Vancouver’s Downtown Eastside *may* be associated with risks among a subset of drug users [49].

Previous studies have shown that although homelessness is not predictive of recidivism [50], unstable housing can contribute to criminogenic risk [51]. Experimental evidence in Vancouver demonstrated that supported housing resulted in a significant decrease in convictions compared to usual care for people experiencing homelessness and mental illness [52].

A significantly higher level of financial support was observed among women, older participants, and those with previously diagnosed substance use and concurrent disorders. In addition, program completion was associated with a greater increase in support. These findings indicate that all participants, *regardless of program completion*, gained access to financial supports that endured at least one year following involvement with the DTCV. Further research is required to further examine the interaction between graduation status and DTCV involvement.

Recent conceptualizations of the Risk-Need-Responsivity (RNR) model - the most influential model of offender treatment [53, 54] - have encouraged the consideration of broader social determinants of health [23]; recognizing that recidivism can be powerfully influenced by factors such as unstable housing, mental illness, lack of pro-social employment, and social exclusion. This broad view appears to be reflected in the current results, whereby the previously reported reductions in recidivism were associated with significant changes in health and social supports.

Relative to the general offender population, the subpopulation of offenders directed to the DTCV appears to include the disproportionate involvement of substance use and other mental disorders, poverty and joblessness, precarious housing, and homelessness [38, 39]. While previous research has established that although mental disorders, per se, are not consistently associated with recidivism, criminogenic risk increases significantly when mental illness co-occurs with a substance use disorder [18, 44]. In our sample, offenders with concurrent disorders were associated with significantly higher rates of health and social service involvement.

### Implications for practice

Our finding that fewer than two-thirds of DTCV participants had been previously diagnosed with substance dependence in the public healthcare system is particularly alarming, and indicates a need to strengthen the involvement of at-risk offenders with community addictions services – prior to committing offences. We found that the presence of concurrent mental illness influenced engagement with services. Thorough assessment for co-occurring mental illness may be a valuable component of DTC practice, setting the stage for integrated treatment. DTCs have been referred to as a “potential bridge…between justice and broader health service systems” ([55]; p.241). In order for DTCs to fulfill this liaison role to its full potential, it is essential that adequate investments are made in the ‘broader health service systems’ required by program participants, including housing, employment, and specific health services. Without these investments offenders remain at risk for recidivism.

### Implications for DTC policy

In order to improve overall DTC success rates, policy makers must advocate for the provision of resources that are responsive to the risks and needs of offenders. Our results suggest that engagement with health and social services contributes to positive DCT outcomes, and that for individuals with complex needs, concerted policy making is indicated. This work requires the leadership of public servants, to recognize inter-dependencies between different program areas for high-risk individuals, and to promote the business case for coordinated approaches exemplified by DTCs.

### Limitations, strengths & future research

Several limitations of this study should be acknowledged, with implications for future research. Although our analyses drew on longitudinal administrative data which provide an objective record of events as they occurred in the lives of DTCV participants (i.e., health services, social assistance benefits and contacts with the criminal justice system), our sole reliance on these data may mask sources of error; such as coding errors, error in gender recording, incompleteness, or inaccuracies associated with professional judgment. For example, the diagnosed prevalence of substance use and mental disorders among offenders is likely to reflect both false positive and false negative errors. These data also omit potentially important forms of service, such as those administered by private psychologists and psychiatrists, although the vast majority of DTCV participants would obtain their health care through the publicly insured health system.

Our study design allowed for only 12 months of participant follow-up. Substance use and recidivism have been linked with improvements in functioning in several domains, emphasizing “…the need to focus on the long-term interplay of multiple events … over time*”* ([56]; p.252). We highly recommend that future research should examine longer follow-up periods with further attention to the interactions between variables observed in our study, including formal education, age, and other socio-demographic characteristics.

This study used an observational cohort design. The absence of a comparison group and the use of a non-experimental design restrict our ability to infer causal relationships between health and social service use and previously published evidence of reduced recidivism within the DTCV cohort [38]. The inclusion of information from sectors beyond justice may therefore be important to understanding offender rehabilitation, particularly with programs such as DTCs, which aim to reduce recidivism by attending to social determinants of both health and public safety. Additional research is also needed to establish the generalizability of the present study to other regions, settings and demographics. While the current research may be highly relevant to DTC participants residing in Vancouver’s Downtown Eastside, the findings may be less generalizable to settings with differing client profiles (e.g., fewer women and Aboriginals) or models of public service (non-universal coverage).

Strengths of the current study include the use of an intent-to-treat approach with a relatively large sample of individuals spanning 12 months prior to DTCV entry and 12 months following exit from the program, regardless of program status at the time of exiting. While abundant research has been published in recent years concerning reductions in recidivism as a result of DTC engagement, *very few studies have extended their focus beyond recidivism as an outcome*, and particularly within the Canadian literature. By examining changes in health and social service involvement, our results are among the first to indicate the potential importance of health and social services as contributors to the rehabilitation of DTC offenders.

## Conclusion

Our primary results show that within our sample of DTCV participants, engagement with community (outpatient) health and social services increased significantly post-program, regardless of offender status at completion. Our findings also demonstrate that women and participants diagnosed with mental disorders or concurrent disorders exhibited significantly higher levels of health and social service use.

These findings affirm the importance of considering the broader social determinants of health in offender rehabilitation and crime reduction initiatives. While future evaluations of DTCs are needed that further elucidate the links between intervention components and primary outcomes such as recidivism, broader social determinants of health – including substance use involvement and high number of days spent in hospital - should be *well identified* prior to referral to drug treatment court, in order to provide the greatest opportunities for success. Proactive measures such as these may contribute to cost avoidance, to the extent that such patterns are attenuated following DTC participation.

## Endnotes

^a^Socio-demographic data for our study, including “self-reported” gender, were obtained from Ministry of Justice data at the date of first sentence. We refer to this variable as “gender”, as we are unable to specify in each case whether it refers to a social or biological construct.

^b^Including admissions to emergency departments, urgent care centres and intensive care. ^c^Including all indigenous people of Canada (i.e., status Indians, non-status Indians, Métis, and Inuit people).

## Abbreviations

ARR: adjusted rate ratio
BC: British Columbia
CAD$: Canadian dollars
DTC: drug treatment court
DTCV: drug treatment court of Vancouver
GEE: generalized estimating equation
ICD: International Classification of Diseases
MSP: medical services plan
NBR: negative binomial regression
NSMD: non-substance related mental disorder
RNR: risk need responsivity
RR: rate ratio
SUD: substance related mental disorder
